# Supplementary material for: Transcriptomic Analysis of the Response of the Toxic Dinoflagellate Prorocentrum lima to Phosphorous Limitation
Source: Microorganisms. 2023 Aug 31;11(9):2216. doi: 10.3390/microorganisms11092216 (PMC10535992; doi:10.3390/microorganisms11092216)
Supplement: Supplementary file 1 [file microorganisms-11-02216-s001.zip › Supplementary Information.pdf]

Transcriptomic Analysis of the Response of the Toxic Dinoflagellate *Prorocentrum lima* to Phosphorous Limitation

Xiukun Wan, Ge Yao, Kang Wang, Yanli Liu, Fuli Wang and Hui Jiang *

State Key Laboratory of NBC Protection for Civilian, Beijing 102205, China; xiukunwan@126.com (X.W.); bzyaoge@163.com (G.Y.); yiyongjun1949@163.com (K.W.); liuh306@hotmail.com (Y.L.); wangfuli5728@163.com (F.W.)

* Correspondence: jiangtide01@sina.cn

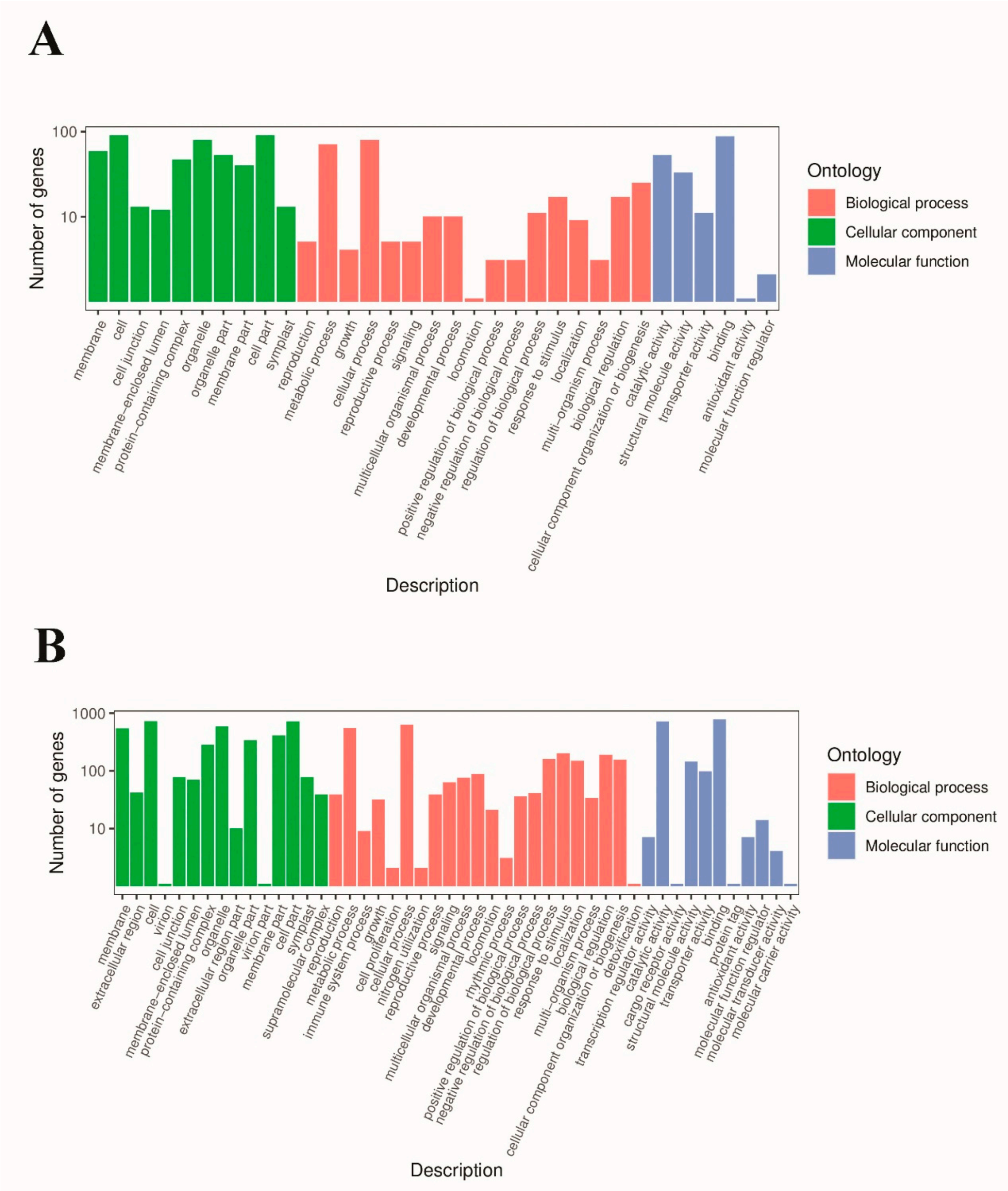

Figure. S1. Differential expression analysis using Gene Ontology (GO) clustering in *Prorocentrum lima* under different P conditions. (A) Statistics of GO enrichment between 1/4 P-limited and P-replete conditions. (B) Statistics of GO enrichment between 1/16 P-limited and P-replete conditions. The horizontal axis represents the multiple biological processes of differentially expressed genes, while the vertical axis represents the number of genes involved in the biological processes.

Table. S1. Descriptions of the differentially expressed genes in *P. lima* under different P conditions with function annotations from KEGG, NR, Swiss-Prot, Tremble, KOG, GO, and Pfam database.

Table. S2. RT-qPCR validations of differentially expressed genes in in *P. lima* under 1/16 P-limited condition.
