# Supplementary material for: Transcriptomic Analysis of the Response of the Toxic Dinoflagellate Prorocentrum lima to Phosphorous Limitation
Source: Microorganisms. 2023 Aug 31;11(9):2216. doi: 10.3390/microorganisms11092216 (PMC10535992; doi:10.3390/microorganisms11092216)
Supplement: Supplementary file 1 [file microorganisms-11-02216-s001.zip › Table S2.pdf]

Table S2. RT-qPCR validations of differentially expressed genes in in *P. lima* under 1/16 P-limited condition.

qPCR				RNA-Seq
Genes	Forward(5′—3′)	Reverse (5′—3′)	log2 (fold change) (1/16P vs 1P)	log2 (fold change) (1/16P vs 1P)
PsbA	GCCTGTTGCTGGGTCAATTC	GCAACAGGAGCTGAAAAGGC	-2.05	-2.94
PsbC	TGGGCAGGGTCAATGTGTTT	CACCTCCTGAAGATACGCCC	-0.94	-3.30
PetD	GCGAGCCTTCAGGTATTGGT	AAGAGAGCAACCTCCAAGCC	-3.18	-3.59
MCAT	GCTACCCCAGACCTTTGCAT	CGTGCAAGCGATGATCAAGG	0.56	1.16
ABCB1	TTCTTCGCTATCCTGGGGGA	CAAGATTGGCGATGCGGAAG	1.24	1.81
